# Daytime Sleepiness from Preschool Children’s and Parents’ Perspectives: Is There a Difference?

**DOI:** 10.3390/children11050568

**Published:** 2024-05-08

**Authors:** Eugenija Marušić, Linda Lušić Kalcina, Ivana Pavlinac Dodig, Zoran Đogaš, Maja Valić, Renata Pecotić

**Affiliations:** 1 Department of Pediatrics, University Hospital of Split, 21000 Split, Croatia; emarusic@kbsplit.hr; 2 Department for Neuroscience, University of Split School of Medicine, 21000 Split, Croatia; llusic@mefst.hr (L.L.K.); ivana.pavlinac@mefst.hr (I.P.D.); zdogas@mefst.hr (Z.Đ.); maja.valic@mefst.hr (M.V.)

**Keywords:** sleepiness, preschool children, Epworth sleepiness scale, sleep

## Abstract

This cross-sectional study investigated the level of daytime sleepiness and sleep-related behaviors in preschool children and compared their self-evaluations with the evaluations of their parents. It was conducted in Split-Dalmatian County, Croatia, among 196 preschool children aged 6–7 years seen at regular medical examinations, accompanied by their parents, using the Epworth sleepiness scale for children and parents/caregivers. Compared to their child’s reports, parents tended to underestimate their child’s sleepiness while sitting in a classroom at school (*p* = 0.001) and overestimate their child’s sleepiness when lying down to rest or nap in the afternoon (*p* < 0.001). Boys were sleepier while sitting in a classroom at school during the morning than girls (*p* = 0.032). As much as 48.2% of preschool children had their own cellphones/tablets. Boys used video games (*p* < 0.001) and cellphones/tablets more than girls did (*p* = 0.064). Parental estimation of children playing video games at bedtime was lower than the child’s report (*p* < 0.001). Children who had a TV in their bedroom reported more daytime sleepiness (*p* = 0.049), and those who played video games at bedtime went to sleep later during the weekend (*p* = 0.024). Also, children owning cellphone/tablets had longer sleep latency during the weekend compared to children not owning a cellphone (*p* = 0.015). This study confirmed that parents tend to underestimate children’s habits of playing video games at bedtime and children’s sleepiness during morning classes. Preschool children who use electronic devices at bedtime more frequently have prolonged sleep latency. These findings provide further evidence of the effects of electronic media devices on preschoolers’ sleep patterns and daytime sleepiness.

## 1. Introduction

It has been estimated that almost 50% of children are likely to experience sleep problems as they grow up [1]. Sleep problems are also prevalent in preschool-aged children and are usually related to prolonged sleep onset latency, frequent night waking, nightmares and difficulty waking in the morning [2]. Previous studies have indicated that insufficient sleep has numerous consequences, including excessive daytime sleepiness, emotional and behavioral problems, cognitive deficits, lower school performance, reduced immune system activity, obesity and metabolic dysfunction [3]. Moreover, sleep disorders such as insomnia, sleep apnea, parasomnias and restless legs syndrome are relatively common among preschool-aged children [4]. In addition, reduced sleep quality resulting from poor sleep hygiene, bedtime difficulties, extended sleep onset latency and nighttime fears may contribute to daytime sleepiness in preschool children. Excessive daytime sleepiness appears to be the most prominent feature of inadequate sleep, and the prevalence of sleepiness in children and adolescents is almost 40% [5]. However, young children might have difficulty expressing their sleep-related symptoms due to the developmental stage, whereas adolescents might minimize or dismiss sleep-related issues [5,6]. The manifestations of excessive daytime sleepiness in children and adolescents can vary, evolving with age and appropriate sleep requirements. Adolescents often face additional social pressures and lifestyle factors that can affect their sleep patterns. Furthermore, the onset of puberty may lead to delayed sleep phase syndrome, causing adolescents to have difficulty falling asleep, whereas younger children typically require more total sleep time, with recommended sleep durations gradually decreasing as they enter adolescence [7,8]. Implementing behavioral interventions customized to the child and family can effectively manage sleepiness. One might conclude that it is essential to evaluate and recognize sleep problems in preschool children to promote optimal development and overall health [3,9]. Inconsistency is present regarding the association between sex and sleep-related difficulties, especially in children [7]. Among the variables associated with short sleep duration in childhood, the use of electronic devices before bedtime and the presence of TV and electronic devices (computers, tablets) in a child’s bedroom negatively affect sleep quality, prolong sleep onset and reduce sleep duration [2,10,11,12,13,14].

A robust and consistent correlation was observed between using light pollution resources such as media devices before bedtime and reduced sleep duration, sleep quality and daytime sleepiness [15]. In Western countries, evidence indicates an association between light pollution from electronic media use and sleep duration, with more robust findings observed in children aged 6–15 years than in those aged 0–5 years [16]. Within the Chinese population, the duration of light pollution from TV viewing and computer and mobile phone usage revealed that excessive use of screen-based electronic devices was predictive of lower sleep quality [17]. In a recent investigation into the sleep habits of children aged 2–5 years, it was found that bright light exposure and computer or mobile use before bedtime increase sleep disturbances and poor sleep hygiene in preschool children [18].

There are various instruments available to assess excessive daytime sleepiness in different age groups of children. The Epworth Sleepiness Scale for Children and Adolescents (ESS-CHAD) is a highly valid, reliable and unidimensional tool for evaluating daytime sleepiness that has been translated and validated in several languages [19,20,21,22]. However, it has yet to be utilized in Croatian children. Factors such as variations in self-assessment and assessments by parents or caregivers might constrain the applicability of the ESS-CHAD in children. Notably, discrepancies can arise between children’s self-evaluated scores and those provided by parents/caregivers on the ESS-CHAD. Assessing the psychometric properties of the ESS-CHAD can aid in establishing precise scoring criteria for children, and more evidence that directly addresses the inquiry regarding age-specific scoring criteria for the ESS-CHAD in children younger than 12 years of age is needed. More evidence might address the inquiry regarding age-specific scoring criteria for the ESS-CHAD in children aged 6–8 years.

In recent reviews from the pediatric sleep literature, it has been emphasized how important healthy sleep is among children of different age groups [5,6]. Among a lot of contributing factors that might be related to children’s sleep health, the use of screen-based media is an important challenge, prompting the reasonable notion of reducing their usage before bedtime to achieve healthy bedtime habits, since excessive daytime sleepiness might come as a consequence of children’s poor bedtime routines [23]. Furthermore, depending solely on parental reports of children’s daytime sleepiness may not offer a comprehensive understanding since parents can only report specific changes in children’s behavior. Incorporating children’s own reports becomes essential for better identification of symptoms such as sleepiness, which could stem from inadequate sleep quality. By examining both children’s self-evaluation of daytime sleepiness and parental assessment, our study aims to shed light on the discrepancy, if any, between their perceptions.

Therefore, the objective of this study was to assess the level of daytime sleepiness among preschool children in Croatia. We aimed to compare the self-reported sleep habits and daytime sleepiness of preschool children with those of their parents. Additionally, we sought to investigate the influence of sex and certain sleep-related behaviors, such as bedtime routines, wake-up times, weekday and weekend sleep durations and the use of electronic devices before bedtime, on daytime sleepiness.

## 2. Materials and Methods

This cross-sectional interview study was conducted in Split-Dalmatia County of Croatia among 196 preschool children aged 6–7 years who were seen at regular medical examinations, accompanied by their parents, performed by school medicine specialists in two school medical clinics providing health care of eight elementary schools in Split-Dalmatia County during spring time season. Preventive medical examination is a prerequisite for primary school enrollment at six years of age according to the Child Health Care System of Croatia [24]. The ESS-CHAD was used to assess and compare the level of daytime sleepiness of children reported by children and their parents. Additional questions were administered to preschool children and their parents about daytime/lifestyle activities associated with the propensity to fall asleep. We collected data about chronic diseases from our sample, and among 196 children, 5 were treated for chronic diseases, using antiepileptic drugs (AEDs) (3 children), insulin (1 child) and antihistamines (1 child). The study was conducted between 20 April 2022, and 30 September 2022. This study was reviewed and approved by the Biomedical Ethics Committee of the University of Split School of Medicine, Split, Croatia. Informed consent from parents or legal guardians for study participation was obtained.

### 2.1. Study Instrument

The Epworth sleepiness scale was originally developed for adults [25] and, as such, was previously translated and validated for the Croatian adult population of sleep clinic patients [26]. Due to some specificities, some adaptations had to be made to administer the questionnaire among children of different age groups. Created for the English-speaking population, the ESS-CHAD has been translated and proven for its reliability and validity in different languages [20,21,27].

The ESS-CHAD is composed of 8 items evaluating the propensity of individuals to fall asleep during a particular set of activities over the past month using a 4-point Likert scale ranging from 0 (never fall asleep), 1 (slight chance of falling asleep), 2 (moderate chance of falling asleep), to 3 (high chance of falling asleep). The parent/caregiver ESS-CHAD is adopted to estimate the child’s likelihood of falling asleep in the same eight circumstances by the parent/caregiver. In both instruments, all activities are evaluated identically, summed and presented as the total ESS-CHAD score, which ranges from 0 to 24. Higher scores indicate more significant daytime sleepiness, as anticipated by the child or parent/caregiver. However, further evidence is yet to be provided for a clear conclusion regarding the range of expected results and excessive daytime sleepiness in adults and children.

The ESS-CHAD was translated from its original English version via instructions published on the official Epworth Sleepiness Scale webpage (https://epworthsleepinessscale.com; accessed on 1 June 2022). The translation into the Croatian language was performed by psychologists, three clinicians and members of the Department of Neuroscience University of Split School of Medicine. The translated version of the ESS-CHAD in the Croatian language was administered to 10 children and their parents at the Department of Neuroscience of the University of Split School of Medicine. The aim was to ensure the face validity and clarity of all the translated items. Back-translation was performed from Croatian into English by a bilingual translator, a native English speaker. The comparison of the English language back-translation of the ESS-CHAD with the original version of the ESS-CHAD was performed by a clinician who was not included in the first translation of the questionnaire. The assessment of items in both versions revealed a similar sentence structure. Finally, a license was provided to use the Epworth Sleepiness Scale for Children and Adolescents Version 2.0 (ESS-CHAD) and the ESS-CHAD parent-caregiver on the Croatian language SPECIAL (TERMS No 78739—22 November 2022 Mapi Research Trust, 2022). The Cronbach’s alpha was 0.7 for the parent/caregiver ESS-CHAD version and 0.6 for the ESS-CHAD version in children. In addition, the parent/caregiver ESS-CHAD version had a test–retest reliability of 0.7.

### 2.2. Procedure

Following a medical examination by a school medicine specialist, the ESS-CHAD questionnaire was distributed to 196 children (88 boys and 108 girls) during the medical/physical examination, which is obligatory for all children before the beginning of primary school in Croatia. The children who answered the ESS-CHAD questionnaire required a medical doctor’s assistance because not all of them knew how to read and write. According to Croatian regulations, parental consent was needed even though the questionnaires were anonymized. If parents allowed their children to participate in the research and decided to join the study, they completed the ESS-CHAD parent/caregiver version of the questionnaire.

Other information regarding lifestyle and sleep habits (e.g., the average number of sleep hours per night; usual bedtime and wake time during weekdays and weekends and/or holidays; the use of a clock alarm or other techniques for waking up a child; methods used to help the child fall asleep; whether the child has a habit of daytime sleep at home or in kindergarten; whether the child owns tablets and mobile phones; the average use of tablets and mobile phones during the day; the use of PlayStation, Xbox or similar video-game devices per day; whether the child plays video games before sleep; TV in the child bedroom; watching TV before sleep; and playing games on tablets, computers or mobile phones before sleep) was obtained from both the children and the parent/caregiver independently from each other.

### 2.3. Data Analysis

All of the analyses were carried out using JASP (JASP 2022 Version 0.16.2) and SPSS (IBM SPSS statistics 2020 for Windows, version 27. Armonk, NY, USA: IBM Corp.). Calculation of the sample size was performed in MedCalc version 11.5.1.0 (MedCalc Software, Mariakerke, Belgium) with the use of a sample size calculator for paired samples, where a mean difference and a standard deviation of differences was calculated based on previous values of the pilot sample, and a minimum required number of paired data was calculated. Differences between categorical (dichotomous) variables were assessed with the chi-square test. Data for continuous and ordinal variables are presented as the mean and standard deviation or as the median and interquartile range, depending on the distribution of the results for a specific variable. When appropriate, nonparametric Mann–Whitney tests of independent groups or Student’s t tests are shown for independent samples, in numerical variables. For the data collected with ESS-CHAD version for children and parents, when asymmetric data distribution was assessed following a significant Kolmogorov–Smirnov test, a decision to use parametric tests was made taking into account the sample size and a visual inspection of Q-Q plots of asymmetrically distributed data. When parental and child responses were compared for paired samples on the ESS, a t-test for paired samples was used. Based on parental assessment of children’s sleep latency duration, three categories were defined (Supplementary Tables S1 and S2) as follows: (1) ≤10 min; (2) 11–20 min; (3) >20 min. For the assessment of the test–retest reliability, baseline and follow-up values (undertaken between three to four weeks) were compared for both children and parents for the final ESS score. Test–retest correlations for each item and the final score were calculated with the Spearman correlation coefficient. Statistical significance was set at *p* < 0.05.

## 3. Results

A total of 196 preschool children, 88 girls (45%) and 108 boys (55%), participated in this study and were assessed in person for their sleep habits and daytime sleepiness with the assistance of a medical doctor during a medical examination. 

### 3.1. Sex Difference Related to Daytime Sleepiness in Preschool Children

Regardless of sex, both girls and boys reported similar values of daytime sleepiness estimated with the ESS-CHAD (Table 1, *p* = 0.807). In addition, there was no significant difference between girls’ and boys’ evaluations of sleepiness during all regular daytime activities, except for more frequent reports of boys regarding sleepiness while sitting in a classroom at school during the morning (0.4 ± 0.8 vs. 0.2 ± 0.5; *p* = 0.032, Table 1). 

### 3.2. The Differences between Children’s and Parents’ Perception of Daytime Sleepiness in Preschool Children

When evaluated by parents who independently estimated their child’s sleepiness using the ESS-CHAD parent/caregiver questionnaire, parents tended to underestimate their child’s sleepiness while sitting in a classroom at school during the morning (E3) compared to their child’s sleepiness (0.2 ± 0.6 vs. 0.3 ± 0.7, *p* = 0.003, Table 2). However, parents tended to overestimate children’s sleepiness while lying down to rest or nap in the afternoon (E5) compared to children’s sleepiness (1.1 ± 1 vs. 0.7 ± 0.9, *p* < 0.001; Table 2).

### 3.3. The Difference between Children’s and Parents’ Perception of Lifestyle and Bedtime Habits in Preschool Children

A total of 48.2% of preschool children at the age of six had their own cell phones or tablets (Table 3). The results of our study indicate that more preschool boys than preschool girls had video-game consoles (*p* < 0.001, Table 3) and tended to own cellphones or tablets (*p* = 0.064, Table 3). In addition, according to a parent’s estimation, 76.6% of preschool children watched TV at bedtime, which is not different from the percentage of children’s reports (70.9%) (*p* = 0.145, Table 4). However, when asked about playing video games before bedtime, parents underestimated that activity compared to the children’s reports (12.8% vs. 25.6%, *p* < 0.001, Table 4). 

Parents were asked to report their children’s sleep habits (Table 5). According to the parents’ estimation, there was a significant difference in the number of daytime naps between girls and boys; preschool girls had significantly more daytime naps than preschool boys did (*p* = 0.022, Table 5).

Parents were asked to evaluate children’s daytime activities associated with their propensity to fall asleep, including owning a cell phone or tablet, playing digital games and watching TV in their bedroom. The results of our study indicate that, according to the parents’ reports, children owning cellphone or tablet had longer sleep latency during the weekend compared to children not owning a cellphone (21.3 ± 17.2 min vs. 16.6 ± 13.3 min, *p* = 0.015, Table 6). Also, children who had a TV in their bedroom were sleepier during the daytime than preschool children who did not have a TV in their bedroom (5.6 ± 3.6 vs. 3.9 ± 3.3, *p* = 0.013, Table 6). 

**Figure 1:**
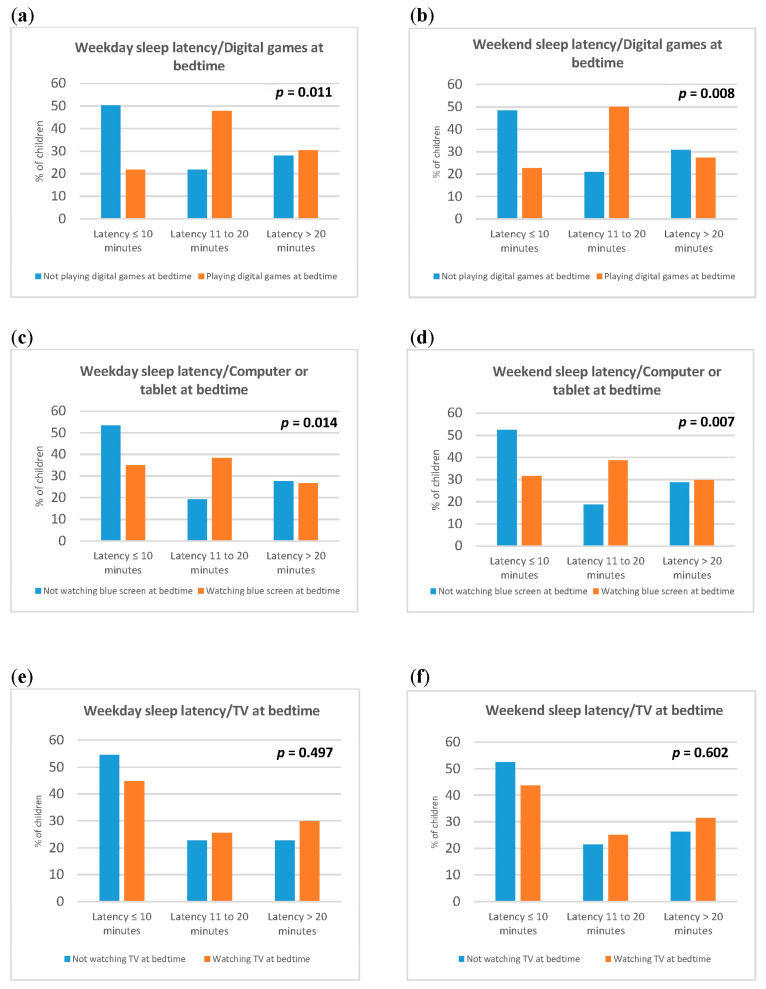
Sleep latency in children during weekdays (**a**,**c**,**e**) and weekends (**b**,**d**,**f**) regarding the use of digital technology at bedtime.

In addition, children who watched TV at bedtime were more sleepy during the day than preschool children who did not watch TV at bedtime were (4.4 ± 3.5 vs. 3.4 ± 3.0, *p* = 0.048, Table 7). When asked about the use of computers or tablets, digital games and TV at bedtime, the results of our study indicate that preschool children who play digital games at bedtime tend to sleep later during the weekend than preschool children who do not play games at bedtime do (22.1 ± 0.8 vs. 21.7 ± 0.6, *p* = 0.004, Table 7). In addition, preschool children who play digital games at bedtime have more frequent prolonged sleep latency both during weekdays (*p* = 0.011) and weekends (*p* = 0.008), as shown in Figure 1. Similarly, preschool children who used computers or tablets at bedtime had more frequent prolonged sleep latency both on weekdays (*p* = 0.014) and weekends (*p* = 0.007), as shown in Figure 1.

## 4. Discussion

The current study revealed that parents, when independently evaluating the child’s sleepiness using the ESS-CHAD parent/caregiver questionnaire, tend to underestimate their child’s sleepiness at preschool and overestimate sleepiness during afternoon rest at home. Regarding the overall assessment of daytime sleepiness in children, the study revealed similar values of daytime sleepiness in both girls and boys aged between 6 and 7 years. The evaluation of sleepiness was also comparable between boys and girls during eight regular daytime activities, as defined by the ESS-CHAD questionnaire. Nevertheless, the study provides strong evidence that boys use electronic devices more than girls do among preschool children. In addition, children with a TV in their bedroom who use cellphones or tablets, digital games and TV before bedtime are recognized as reporting more pronounced daytime sleepiness.

In a recent review, sleepiness was described as being awake but having a heightened inclination to fall asleep, whereas excessive daytime sleepiness (EDS) is “a subjective feeling of drowsiness or an increased propensity to fall asleep, occurring during periods and in circumstances when an individual would typically be awake and alert” [5]. Among preschool-aged children and early adolescents, EDS can stem from diverse factors, including sleep deprivation, sleep disorders and social or behavioral influences [28,29]. EDS in children is frequently overlooked or underestimated because parents tend to underreport it, physicians often fail to diagnose it accurately and preschool children are not aware of the severity of sleepiness for their wellbeing [5]. Our study revealed a significant link between daytime sleepiness and inadequate sleep habits, primarily related to the bedtime routine before sleep. According to the results of our study, the use of TV in the bedroom has been strongly linked to daytime sleepiness, whereas watching television and playing video games prior to sleeping were associated with later bedtimes. These results are in accordance with those of several previous studies showing that screen-based media, which are increasingly present in children as early as they are in preschool children, impact children’s sleep quality and well-being [15]. A recent systematic review revealed a lack of clear associations and consistency regarding the correlates of mobile screen usage (including mobile phones, electronic tablets or computers) among children aged 0 to 8 years. The challenge arises from segregating study findings across various types of mobile screen media, rendering the results predominantly descriptive rather than providing a cohesive pattern [30]. Findings from a systematic review by Carter et al. reported that using devices before bedtime is correlated with a greater likelihood of insufficient sleep duration, diminished sleep quality and profound daytime sleepiness [15]. Furthermore, merely having media devices in the bedroom, regardless of usage, was linked to a greater probability of negative sleep consequences among children and adolescents between 6 and 19 years of age [15]. Our study contributes to such findings with the inclusion of preschool-aged children, adding to the growing body of evidence suggesting detrimental effects of the aforementioned device usage habits on sleep quality. Additionally, the current study provides evidence on the lack of parental awareness of children’s habits of playing games before sleeping, as well as a lack of awareness of children’s sleepiness in certain situations, even among parents of children as young as 5 or 6 years of age.

The methodology employed in this research entails the evaluation of daytime sleepiness using structured questionnaires, specifically the ESS-CHAD designed for children and the ESS-CHAD for parents/caregivers, representing the predominant approach for examining sleepiness in young children. The ESS-CHAD has undergone validation in various populations, including Turkish children aged 12–18 years and Australian adolescents aged 12–18 years, demonstrating strong internal consistency, test–retest reliability and construct validity [27,31]. The ESS-CHAD was adapted from the adult Epworth Sleepiness Scale to tailor the experiences of children and adolescents better and, as such, might be used in the pediatric population. Therefore, adjustments were made to use age-appropriate language and to reflect activities relevant to a population of children [32]. Previous research provided evidence that most children over seven years and adolescents can complete the ESS-CHAD independently [27]. However, the assistance of parents/caregivers or medical experts is needed to assess younger children’s sleepiness, as was necessary in our study that investigated preschool children [27,32]. Conducting further research with younger children could help address the challenges of recognizing symptoms and understanding scales inherent in this population. Investigating the feasibility and reliability of using the ESS-CHAD with children under 7 years would be an important step in improving our understanding of daytime sleepiness in younger children and enhancing the assessment tools available for this age group.

Due to the study’s cross-sectional design, we cannot make long-term conclusions solely from the presented results. However, even though causality cannot be definitively established, our findings suggest the potential benefits of intervention in increasing public awareness, particularly among children and parents, regarding the use of electronic devices before sleep. Additionally, this study is constrained by its dependence on subjective child- or parent-reported results. While parents’ evaluations of children’s sleep habits yield acceptable results, they might be less reliable than objective assessments providing correct estimations of sleep onset, continuity of sleep or disruption of sleep. With regard to the child’s age, the help of medical doctors who aid in the collection of the child’s reports on daytime sleepiness must be considered. Factors influencing scores on the Epworth Sleepiness Scale in children may include obstructive sleep apnea, epilepsy, AED, antihistamines and other medical conditions, such as parasomnias, narcolepsy and hypersomnia [5,33]. Since our study included only five children who were using drugs for chronic diseases (2.5%), the outcomes of the study were not substantially affected. Also, future studies may benefit from including assessments of caffeine intake to better understand its relationship with sleep quality and daytime sleepiness in this age group.

## 5. Conclusions

In conclusion, when parents and children independently assessed children’s sleep habits and daytime sleepiness using the ESS-CHAD questionnaire, there were no differences in the assessment of children’s daytime sleepiness, except for parents underestimating the children’s sleepiness during the morning classes and overestimating sleepiness when lying down to rest or nap in the afternoon. Regarding the estimation of sleep habits, parents tended to underestimate children’s habit of playing video games at bedtime. Since almost 50% of children at the age of six had their own cell phones or tablets, with electronic devices being more commonly owned by preschool boys than girls, it is important for parents to understand the impact of electronic devices on sleep habits. For instance, watching TV, a prevalent bedtime routine among preschool children, was associated with more pronounced daytime sleepiness. Additionally, preschool children who use electronic devices at bedtime more frequently have prolonged sleep latency. This study provides further evidence on the effects of electronic media devices on preschoolers’ sleep patterns and daytime sleepiness. This finding underscores the necessity for proactive intervention, such as educating children from an early age and their parents to exclude electronic devices at bedtime, promoting better sleep quality and reduced daytime sleepiness.

## Figures and Tables

**Table 1:** Daytime sleepiness assessed by boys and girls.

	Boys (*n* = 108)	Girls (*n* = 88)	*p* *
ESS-CHAD total	3.9 ± 3	3.8 ± 2.9	0.807
E1	0.5 ± 0.7	0.5 ± 0.8	0.862
E2	0.7 ± 0.9	0.7 ± 0.8	0.934
E3	0.4 ± 0.8	0.2 ± 0.5	0.032 *
E4	1.1 ± 1.1	1.1 ± 1.1	0.956
E5	0.6 ± 0.9	0.7 ± 0.9	0.753
E6	0 ± 0.2	0.1 ± 0.2	0.687
E7	0.4 ± 0.7	0.5 ± 0.8	0.471
E8	0 ± 0.2	0 ± 0.2	0.644

ESS-CHAD: Epworth Sleepiness Scale for Children and Adolescence; E1–8: Eight items of the ESS-CHAD. Data are presented as mean ± standard deviation. * Student’s t-test for independent samples.

**Table 2:** Comparison of daytime sleepiness assessed by parents and children.

	Parents (*n* = 196)	Children (*n* = 196)	*p*
ESS-CHAD total	4.1 ± 3.4	3.9 ± 2.9	0.261
E1	0.4 ± 0.8	0.5 ± 0.7	0.232
E2	0.8 ± 0.9	0.7 ± 0.9	0.390
E3	0.2 ± 0.6	0.3 ± 0.7	0.003 *
E4	1.2 ± 1	1.1 ± 1.1	0.596
E5	1.1 ± 1	0.7 ± 0.9	<0.001 *
E6	0.1 ± 0.3	0.1 ± 0.2	0.835
E7	0.4 ± 0.7	0.5 ± 0.7	0.232
E8	0.1 ± 0.4	0 ± 0.2	0.252

ESS-CHAD: Epworth Sleepiness Scale for Children and Adolescence; E1–8: Eight items of the ESS-CHAD. Data are presented as mean ± standard deviation. * Student’s t-test comparison for paired samples.

**Table 3:** Children’s self-assessment of lifestyle habits in girls and boys.

	Total	Girls (*n* = 88)	Boys (*n* = 108)	*p*
Age	6 (6–7)	6 (6–7)	6 (6–7)	0.699 *
Cell phone or tablet owner	94 (48.2%)	36 (40.9%)	58 (54.2%)	0.064 **
Owner of video-games consoles	59 (30.7%)	11 (12.8%)	48 (45.3%)	<0.001 **

Age is presented as median (interquartile range) and data about lifestyle habits are presented as total number (percentage). * Mann–Whitney comparison for independent samples. ** Pearson chi-square.

**Table 4:** Lifestyle habits at bedtime estimated by children and their parents.

	Parents (*n* = 196)	Children (*n* = 196)	*p* *
Do you play video-games at bedtime?	25 (12.8%)	50 (25.6%)	<0.001
Do you watch TV at bedtime?	151 (76.6%)	139 (70.9%)	0.145

Data are presented as total number (percentage). * Wilcoxon signed ranks test.

**Table 5:** Parental assessment of children’s sleep habits.

		Total (*n* = 196)	Girls (*n* = 88)	Boys (*n* = 108)	*p* *
Total sleep time (h)		9.7 ± 1	9.7 ± 0.9	9.7 ± 1	0.927
Bedtime (h)	Weekday	21.2 ± 0.7	21.3 ± 0.6	21.2 ± 0.7	0.538
	Weekend	21.7 ± 0.7	21.7 ± 0.6	21.7 ± 0.7	0.698
Waketime (h)	Weekday	7.5 ± 0.7	7.6 ± 0.8	7.4 ± 0.7	0.190
	Weekend	8.2 ± 0.8	8.3 ± 0.7	8.2 ± 0.9	0.225
Sleep latency (min)	Weekday	17.8 ± 14.5	17.8 ± 11.7	17.7 ± 16.5	0.972
	Weekend	19.4 ± 16.9	19.9 ± 14.2	19 ± 18.8	0.698
Daytime naps (per week)		4.3 ± 1.8	4.8 ± 1.8	3.8 ± 1.9	0.022

Data are presented as mean ± standard deviation. * Student’s t-test for independent samples.

**Table 6:** Parental assessment of daytime sleepiness and sleep habits in children.

		Owning Cellphone or Tablet		Playing Digital Games		Having TV in the Child’s Bedroom	
		No (*n* = 145)	Yes (*n* = 51)	No (*n* = 140)	Yes (*n* = 56)	No (*n* = 168)	Yes (*n* = 28)
ESS-CHAD parent/caregiver		4.1 ± 3.4	4.5 ± 3.4	4.2 ± 3.4	4.3 ± 3.4	3.9 ± 3.3 *	5.6 ± 3.6 *
Total sleep time (h)		9.9 ± 1	9.6 ± 1	9.7 ± 1	9.6 ± 1	9.7 ± 0.9	9.4 ± 1.2
Bedtime (h)	Weekday	21.2 ± 0.6	21.3 ± 0.7	21.3 ± 0.7	21.1 ± 0.6	21.2 ± 0.6	21.4 ± 0.8
	Weekend	21.7 ± 0.6	21.8 ± 0.7	21.7 ± 0.7	21.7 ± 0.7	21.7 ± 0.7	21.8 ± 0.8
Waketime (h)	Weekday	7.5 ± 0.7	7.5 ± 0.8	7.6 ± 0.8	7.4 ± 0.6	7.5 ± 0.7	7.6 ± 0.8
	Weekend	8.2 ± 0.8	8.3 ± 1	8.3 ± 0.9	8.1 ± 0.8	8.2 ± 0.8	8.4 ± 0.9
Sleep latency (min)	Weekday	16.6 ± 13.3	21.3 ± 17.2	18.3 ± 14.8	16.3 ± 13.5	17.3 ± 12.9	20.5 ± 22.5
	Weekend	17.6 ± 14 **	24.5 ± 22.5 **	20 ± 18.2	17.9 ± 12.6	18.8 ± 15.9	23.5 ± 22.6
Daytime naps (per week)		5 (5–5)	5 (2.5–5)	2.5 (1.8–3)	5 (3.8–5)	5 (4–6)	5 (3–5)

ESS-CHAD, Epworth Sleepiness Scale for Children and Adolescents; E1–8, Eight items of the ESS-CHAD; * Differences following parametric t-test comparison of independent groups, *p* = 0.013; ** Differences following parametric t-test comparison of independent groups, *p* = 0.015.

**Table 7:** Parental assessment of daytime sleepiness and sleep habits in children using electronics at bedtime.

		Watching Computer or Tablet at Bedtime		Watching TV at Bedtime		Playing Digital Games at Bedtime	
		No (*n* = 126)	Yes (*n* = 63)	No (*n* = 45)	Yes (*n* = 151)	No (*n* = 170)	Yes (*n* = 25)
ESS-CHAD parent/caregiver		4 ± 3.4	4.4 ± 3.4	3.4 ± 3 *	4.4 ± 3.5 *	4.1 ± 3.4	4.5 ± 3.6
Total sleep time (h)		9.7 ± 1	9.8 ± 1	9.7 ± 1.2	9.7 ± 0.9	9.7 ± 1	9.7 ± 0.8
Bedtime (h)	21.2 ± 0.6	21.3 ± 0.8	21.2 ± 0.6	21.2 ± 0.7	21.2 ± 0.6	21.5 ± 0.9	21.4 ± 0.8
	21.6 ± 0.6	21.8 ± 0.7	21.7 ± 0.5	21.7 ± 0.7	21.7 ± 0.6 **	22.1 ± 0.8 **	21.8 ± 0.8
Waketime (h)	7.5 ± 0.8	7.5 ± 0.7	7.6 ± 0.7	7.5 ± 0.8	7.5 ± 0.8	7.6 ± 0.6	7.6 ± 0.8
	8.2 ± 0.8	8.4 ± 0.8	8.1 ± 0.8	8.3 ± 0.8	8.2 ± 0.8	8.4 ± 0.8	8.4 ± 0.9
Sleep latency (min)	16.8 ± 13.7	18.3 ± 13.2	17.6 ± 15.7	17.8 ± 14.1	17.2 ± 14.3	22 ± 15.4	20.5 ± 22.5
	17.3 ± 13.6	21.6 ± 19.4	19.8 ± 21.4	19.3 ± 15.4	18.7 ± 15.4	25.5 ± 24.9	23.5 ± 22.6
Daytime naps (per week)		4 (2–5)	5 (4–5)	5 (4.3–5)	5 (3–5)	5 (2.8–5)	3 (3–3.5)

ESS-CHAD, Epworth Sleepiness Scale for Children and Adolescents; E1–8, Eight items of the ESS-CHAD; Data on ESS, bedtime, waketime and sleep latency are presented as mean ± standard deviation, while daytime naps are presented as median (interquartile range); * Differences following Mann–Whitney test comparison for independent samples, *p* = 0.048; ** Differences following t-test comparison for independent samples, *p* = 0.004.

## Data Availability

All data on which the study is based are available in anonymized form via the corresponding author due to ethical restrictions.

## Supplementary Materials

The following supporting information can be downloaded at: https://www.mdpi.com/article/10.3390/children11050568/s1, Table S1: Frequency of electronic device usage at bedtime in three categories of sleep latency during the weekday; Table S2: Frequency of electronic device usage at bedtime in three categories of sleep latency during the weekend.

## References

[1] Carter K.A., Hathaway N.E., Lettieri C.F. (2014). Common sleep disorders in children. Am. Fam. Physician.

[2] Garrison M.M., Liekweg K., Christakis D.A. (2011). Media use and child sleep: The impact of content, timing, and environment. Pediatrics.

[3] Moreira G.A., Pradella-Hallinan M. (2017). Sleepiness in Children: An Update. Sleep Med. Clin..

[4] Licis A. (2017). Sleep Disorders: Assessment and Treatment in Preschool-Aged Children. Child Adolesc. Psychiatr. Clin. N. Am..

[5] Bruni O. (2023). Approach to a sleepy child: Diagnosis and treatment of excessive daytime sleepiness in children and adolescents. Eur. J. Paediatr. Neurol. EJPN Off. J. Eur. Paediatr. Neurol. Soc..

[6] Meltzer L.J., Williamson A.A., Mindell J.A. (2021). Pediatric sleep health: It matters, and so does how we define it. Sleep Med. Rev..

[7] Lewien C., Genuneit J., Meigen C., Kiess W., Poulain T. (2021). Sleep-related difficulties in healthy children and adolescents. BMC Pediatr..

[8] Ophoff D., Slaats M.A., Boudewyns A., Glazemakers I., Van Hoorenbeeck K., Verhulst S.L. (2018). Sleep disorders during childhood: A practical review. Eur. J. Pediatr..

[9] Vriend J., Davidson F., Rusak B., Corkum P. (2015). Emotional and Cognitive Impact of Sleep Restriction in Children. Sleep Med. Clin..

[10] Calamaro C.J., Yang K., Ratcliffe S., Chasens E.R. (2012). Wired at a young age: The effect of caffeine and technology on sleep duration and body mass index in school-aged children. J. Pediatr. Health Care Off. Publ. Natl. Assoc. Pediatr. Nurse Assoc. Pract..

[11] Nuutinen T., Ray C., Roos E. (2013). Do computer use, TV viewing, and the presence of the media in the bedroom predict school-aged children’s sleep habits in a longitudinal study?. BMC Public Health.

[12] Cespedes E.M., Gillman M.W., Kleinman K., Rifas-Shiman S.L., Redline S., Taveras E.M. (2014). Television viewing, bedroom television, and sleep duration from infancy to mid-childhood. Pediatrics.

[13] Cain N., Gradisar M. (2010). Electronic media use and sleep in school-aged children and adolescents: A review. Sleep Med..

[14] Brambilla P., Giussani M., Pasinato A., Venturelli L., Privitera F., Miraglia Del Giudice E., Sollai S., Picca M., Di Mauro G., Bruni O. (2017). Sleep habits and pattern in 1–14 years old children and relationship with video devices use and evening and night child activities. Ital. J. Pediatr..

[15] Carter B., Rees P., Hale L., Bhattacharjee D., Paradkar M.S. (2016). Association Between Portable Screen-Based Media Device Access or Use and Sleep Outcomes: A Systematic Review and Meta-analysis. JAMA Pediatr..

[16] Lund L., Solvhoj I.N., Danielsen D., Andersen S. (2021). Electronic media use and sleep in children and adolescents in western countries: A systematic review. BMC Public Health.

[17] Xie Y.J., Cheung D.S., Loke A.Y., Nogueira B.L., Liu K.M., Leung A.Y., Tsang A.S., Leong C.S., Molassiotis A. (2020). Relationships Between the Usage of Televisions, Computers, and Mobile Phones and the Quality of Sleep in a Chinese Population: Community-Based Cross-Sectional Study. J. Med. Internet Res..

[18] Abou-Khadra M.K., Ahmed D., Sadek S.A., Mansour H.H. (2022). Sleep patterns, problems, and habits in a sample of Egyptian preschoolers. Sleep Sci..

[19] Imani V., Lin C.Y., Jalilolgadr S., Pakpour A.H. (2018). Factor structure and psychometric properties of a Persian translation of the Epworth Sleepiness Scale for Children and Adolescents. Health Promot. Perspect..

[20] Lee W.Y., Lau M.N., Soh E.X., Yuen S.W., Ashari A., Radzi Z. (2023). Validation of the Malay version of Epworth sleepiness scale for children and adolescents (MESS-CHAD). BMC Oral Health.

[21] Nunes M.L., Kalil Neto F., Hemb M., Halal C. (2022). Translation and language validation of the Epworth sleepiness scale for children and adolescents (ESS-CHAD) into Brazilian Portuguese. Sleep Sci..

[22] Uygun S.D., Bilbay N.T. (2022). Psychometric evaluation of the Turkish adaptation of the Epworth sleepiness scale for children and adolescents. Child Health Care.

[23] Ricci C., Ordnung M., Rothenbacher D., Genuneit J. (2024). Substituting Book Reading for Screen Time Benefits Preschoolers’ Sleep Health: Results from the Ulm SPATZ Health Study. Nat. Sci. Sleep.

[24] Mestrovic J., Bralic I., Simetin I.P., Mujkic A., Radonic M., Rodin U., Troselj M., Stevanovic R., Benjak T., Pristas I. (2016). The Child Health Care System of Croatia. J. Pediatr..

[25] Johns M.W. (1991). A new method for measuring daytime sleepiness: The Epworth sleepiness scale. Sleep.

[26] Pecotic R., Dodig I.P., Valic M., Ivkovic N., Dogas Z. (2012). The evaluation of the Croatian version of the Epworth sleepiness scale and STOP questionnaire as screening tools for obstructive sleep apnea syndrome. Sleep Breath. Schlaf Atm..

[27] Janssen K.C., Phillipson S., O’Connor J., Johns M.W. (2017). Validation of the Epworth Sleepiness Scale for Children and Adolescents using Rasch analysis. Sleep Med..

[28] Fallone G., Owens J.A., Deane J. (2002). Sleepiness in children and adolescents: Clinical implications. Sleep Med. Rev..

[29] Hosokawa R., Tomozawa R., Fujimoto M., Anzai S., Sato M., Tazoe H., Katsura T. (2022). Association between sleep habits and behavioral problems in early adolescence: A descriptive study. BMC Psychol..

[30] Paudel S., Jancey J., Subedi N., Leavy J. (2017). Correlates of mobile screen media use among children aged 0–8: A systematic review. BMJ Open.

[31] Ogutlu H., Uygun S.D., Randler C. (2021). Psychometric Properties of the Turkish version of the Morningness—Eveningness Stability Scale improved (MESSi) in Adolescents. Chronobiol. Int..

[32] Johns M.W. (2015). The assessment of sleepiness in children and adolescents. Sleep Biol. Rhythm..

[33] Fulfs T., Poulain T., Vogel M., Nenoff K., Kiess W. (2024). Associations between sleep problems and emotional/behavioural difficulties in healthy children and adolescents. BMC Pediatr..

