# Supplementary material for: Daytime Sleepiness from Preschool Children’s and Parents’ Perspectives: Is There a Difference?
Source: Children (Basel). 2024 May 8;11(5):568. doi: 10.3390/children11050568 (PMC11120150; doi:10.3390/children11050568)
Supplement: Supplementary file 1 [file children-11-00568-s001.zip › children-2984298-supplementary.pdf]

Table S1. Frequency of electronic devices usage at bedtime in three categories of sleep latency during the weekday

		**Sleep latency**			
		**≤ 10 minutes**	**10 to 20 minutes**	**> 20 minutes**	***p****
**Watching computer or tablet at bedtime**	**No**	64 (53.3)	23 (19.2)	33 (27.5)	0.014
	**Yes**	21 (35)	23 (38.3)	16 (26.7)	
**Watching TV at bedtime**	**No**	24 (54.5)	10 (22.7)	10 (22.7)	0.498
	**Yes**	63 (44.7)	36 (25.5)	42 (29.8)	
**Playing digital games at bedtime**	**No**	81 (50.3)	35 (21.7)	45 (28)	0.011
	**Yes**	5 (21.7)	11 (47.8)	7 (30.4)	

*Chi square test

Table S2. Frequency of electronic devices usage at bedtime in three categories of sleep latency during the weekend

		Sleep latency			
		**≤ 10 minutes**	**10 to 20 minutes**	**> 20 minutes**	***p****
**Watching computer or tablet at bedtime**	**No**	62 (52.5)	22 (18.7)	34 (28.8)	0.007
	**Yes**	18 (31.6)	22 (38.6)	17 (29.8)	
**Watching TV at bedtime**	**No**	22 (52.4)	9 (21.4)	11 (26.2)	0.602
	**Yes**	61 (43.6)	35 (25.0)	44 (31.4)	
**Playing digital games at bedtime**	**No**	77 (48.4)	33 (20.8)	49 (30.8)	0.008
	**Yes**	5 (22.7)	11 (50.0)	6 (27.3)	

*Chi square test
